# Understanding the Implementation of Family-Centered Care in COVID-19 Isolation Rooms Through Pediatric Nurses’ Experiences: Qualitative Descriptive Study

**DOI:** 10.2196/74403

**Published:** 2025-07-24

**Authors:** Siti Wahyuni, Dessie Wanda, Happy Hayati

**Affiliations:** 1 Sardjito Hospital, Sinduadi, Mlati, Sleman, Di Yogyakarta, Indonesia; 2 Faculty of Nursing, University Indonesia, FIK UI Campus, Jl. Prof. Dr. Bahder Djohan, Depok, West Java, 16424, Indonesia, 62 85695180195

**Keywords:** child, COVID-19, family-centered care, nurses, isolation room

## Abstract

**Background:**

Even though the COVID-19 pandemic has passed, the implementation of regulations in pediatric isolation rooms during the COVID-19 pandemic put enormous pressure on the practice of family-centered care (FCC). For nurses in isolation rooms for children with COVID-19, it was a challenge to implement FCC, which is an approach to child health care that supports the strengths of families and increases the involvement of parents to provide the best care outcomes.

**Objective:**

This study aimed to explore nurses’ experiences in implementing FCC in isolation rooms caring for children with COVID-19.

**Methods:**

A qualitative descriptive design was used based on semistructured interviews with 11 nurses who met the inclusion criteria and worked in 1 of 3 isolation rooms caring for children with COVID-19 in a tertiary hospital in Indonesia. Interviews were recorded and transcribed, then analyzed using thematic analysis.

**Results:**

Three themes were generated, including improving psychological well-being, encouraging family involvement, and making arrangements for communication.

**Conclusions:**

Communication was key to the implementation of FCC in pediatric isolation rooms, and it provided positive results, so it should continue to be implemented even after the pandemic has passed.

## Introduction

Isolation rooms for children with COVID-19, and the regulations that were placed on them during the pandemic, were a threat to the implementation of family-centered care (FCC) [1], an approach that facilitates healing by supporting the strengths of families and improving the involvement of parents, thereby providing positive experiences for children [2]. In isolation rooms, only one parent was allowed to wait for the patient, and the patient was not allowed to receive visits, which put them at risk of mental health impacts due to the limited emotional support they received [3]. Restrictions on physical visits to the isolation room can reduce coping and resilience, which can further affect family involvement [4]. Regulations in the isolation rooms were strict, including the use of personal protective equipment (PPE) and limited interaction, leading to work overload [1] and concerns about contracting COVID-19 among nurses. Nurses need strategies for implementing FCC and maintaining family integrity to increase parental support and involvement [5]. FCC is an approach to child health care that emphasizes respectful, collaborative partnerships between health care professionals and families to meet their physical, emotional, social, and developmental needs [2]. The implementation of FCC in COVID-19 isolation rooms became a challenge to nurses during and after the pandemic. The objective of this study was to explore in depth the experiences of nurses in implementing FCC in isolation rooms for children with COVID-19.

## Methods

### Procedure and Participants

This was a qualitative descriptive study. Eleven participants were selected from a total of 42 nurses who cared for pediatric patients with COVID-19 in the isolation rooms of a tertiary hospital using a purposive sampling method. Inclusion criteria were a minimum of 5 years of working in a pediatric ward, 2 months of working in a COVID-19 isolation room, and obtaining an adequate score on a questionnaire testing their knowledge of FCC (minimum score of 60; range 0‐100). The questionnaire was validated using a Pearson product-moment correlation (≥0.514). The reliability test used Cronbach α, with a result of 0.86 [6]. In addition to meeting the inclusion criteria, the participants also met criteria for research sample variation, including age, education, length of work in COVID-19 isolation rooms, length of work in the pediatric ward, and FCC knowledge score; the aim was to provide a deep, accurate understanding of the existing phenomenon [7].

### Ethical Considerations

Ethical approval was obtained for this study from the Medical and Health Research Ethics Committee of Universitas Indonesia (KE/FK/0325/EC/2023). This study followed the ethical principles outlined in the international and national guidelines on ethical standards and procedure for research with human beings. All matters related to research ethics were outlined in the research explanation sheet, including that if a participant experienced any physical or psychological discomfort, they could report this to the researcher, who would turn off the voice recorder and provide the participant with time to rest. The interview and voice recording resumed if the participant gave their consent. Participants had the right to be treated fairly, and the researchers guaranteed their privacy throughout the study. All participant information and identities are kept confidential, and the results of the study are reported using pseudonyms for anonymity. Participants’ identities and names are known only to the researchers and the research supervisor. Participants were allowed to refuse to participate or withdraw from the study at any time without any consequences or sanctions. If any questions made them uncomfortable, they had the right to refuse to answer. Participants received compensation in the form of feedback on the implementation of family-centered care, souvenirs (blankets and mugs), and internet data packages worth US $5 for being contacted via WhatsApp, video call, or Zoom as a token of appreciation for their willingness to participate in the study.

### Collection of Data

Data were collected in 2023 through semistructured interviews using a voice recorder and field notes, and then the data were transcribed. Bracketing and avoidance of leading were used, and the data analysis used thematic analysis [8]. First, the researchers familiarized themselves with the data. Second, they generated initial codes that appeared relevant to the research question. Third, they searched for themes representing significant patterns in the data and started organizing the codes into overarching themes and subthemes. Fourth, the researchers reviewed the themes to ensure that they were coherent and accurately represented the data. Fifth, the researchers defined and named the themes to refine and develop each theme. Finally, the researchers produced the report. Trustworthiness of the data was achieved through credibility, transferability, dependability, and confirmability [7]. To achieve credibility, the researchers built mutual trust with the participants and conducted member checking; transferability was ensured through purposive sampling and recruiting participants with varied characteristics; dependability was accomplished by carrying out a structured data analysis and inquiry audit through consultation with a supervisor as the internal reviewer; and confirmability was attained by peer review of the research results.

## Results

### Overview

All participants had high education and adequate knowledge about FCC, which is essential for professionalism and clinical practice, as well as for implementation of FCC [9]. Participant characteristics are shown in Table 1.

**Table 1.** Participant characteristics (N=11).

Characteristics		Participants, n (%)
Age (years)		
	36‐35	3 (27)
	36‐45	6 (55)
	46‐55	2 (18)
Nursing education		
	3-year diploma	8 (72)
	4-year bachelor’s degree	3 (28)
Length of time working as a nurse (years)		
	≤10	1 (9)
	>10	10 (91)
Length of work in pediatric COVID-19 rooms (years)		
	≤1	6 (55)
	>1	5 (45)
Family-centered care knowledge		
	Enough	6 (55)
	Good	5 (45)

### Themes

This research generated 3 themes, illustrated in Table 2: improving psychological well-being, encouraging family involvement, and making arrangements for communication.

**Table 2.** Themes generated during interviews.

Theme	Description	Illustrative quotes
Improving psychological well-being	Nurses calming children and supporting parents as they calm children	“In the isolation setting, many expressions are not captured...with the PPE, it’s like we’re creating a barrier.” [Participant 11] “We try to listen to them...advice the mother to accompany the child, reassuring the child so they are not afraid.” [Participant 8] “...playing together to reduce trauma to children” [Participant 11] “We explain the child’s condition, support the parents to be patient, and pray.” [Participant 8]
Encouraging family involvement	Involvement was achieved by first providing health education	“We need to work together with parents...so that all plans can be implemented well.” [Participant 10] “Mom can help take medicine, because mom or family knows how to persuade children, right? Whatever it is, we must inform them...nursing plan, procedure, infection prevention...” Participant 11] “Later, if there’s a problem like choking...please tell us.” [Participant 4] “We educate several times because parent don’t immediately understand.” [Participant 10] “...we speak in a language that is easy to understand...” [Participant 11]” “...to be well-received.” [Participant 7]
Making arrangements for communication	Due to the excessive amount of work, communication in the isolation rooms was carried out simultaneously and communication aids were used	“The activities here are busy, so we plan activities simultaneously.” [Participant 7] “Family had already been informed in the early stages of hospitalisation...” [Participant 5] “We use CCTV to monitor patient, a nurse call system to ensure effective communication, a handphone to coordinate logistics fulfillment with the patient’s family...etc” [Participant 3]

## Discussion

Nurses understand that parents and children feel anxiety in the hospital environment and from wearing complete PPE [10], so they strengthen FCC [11] by listening to the parents’ feelings [12], thereby calming them and their children so they can adapt to the hospital environment [4]. Nurses encourage family involvement [13] because the parents are the ones who best understand their children [10] and are most competent in their care; their function as caregivers must therefore continue [14]. Participation of parents reduces anxiety in both children [5] and parents [15], ensuring that the children’s psychological needs are met [16] and facilitating healing [17].

Nurses have previously considered how far they should involve parents in child care [11,18]. Parents need information [19] in language that is easy to understand and comprehend [20], and they must receive this information at an early stage of their child’s hospitalization [3]. If the parents are not trained and educated, procedural errors can occur [16]. Ensuring that families have the same views as health practitioners is also a condition for cooperation [21] and for parents to be involved effectively in decision-making [22]. Moreover, this helps carry out nursing care without mistakes [3].

Communication became a major challenge during the COVID-19 pandemic due to the use of PPE and work overload [1,23]. Masks and face shields reduced the volume and clarity of spoken language, making it difficult to hear soft tones of voice or read facial expressions [16]. Human contact relies on gentle touch [24]. Telecommunication (eg, telephone, video calls, or WhatsApp messages) became a solution that supported emotional conversations between patients, families [25], and health practitioners. It reduced contact between the patient and health practitioner and supported the continuity of health management [3]. The use of closed-circuit television combined with a nurse call system was found to be more effective [26] in facilitating communication in isolation rooms [27]. In addition, it helped family members outside the room to directly see the condition of the child and family member in the isolation room, making them feel calmer [27]. In this study, parents were not interviewed to determine their views on their experiences as compared to those of the nurses, as we only targeted nurses. This may have limited the solidity of our conclusions, and our findings may not be fully generalizable. Nevertheless, we found that communication was the key to implementing FCC, and it provided positive results in pediatric isolation rooms, so it should continue to be implemented even after the pandemic has passed. Institutional support is needed to develop nurses’ communication.

## Abbreviations

FCC: family-centered care
PPE: personal protective equipment
